# Characterization of Mental Health in US Veterans Before, During, and 2 Years After the Onset of the COVID-19 Pandemic

**DOI:** 10.1001/jamanetworkopen.2023.0463

**Published:** 2023-02-23

**Authors:** Ian C. Fischer, Peter J. Na, Ilan Harpaz-Rotem, John H. Krystal, Robert H. Pietrzak

**Affiliations:** 1 National Center for Posttraumatic Stress Disorder, VA Connecticut Healthcare System, West Haven, Connecticut; 2 Department of Psychiatry, Yale School of Medicine, New Haven, Connecticut; 3 VA Connecticut Healthcare System, West Haven, Connecticut

## Abstract

This cohort study of US veterans reports changes in rates of anxiety and depressive disorders following the COVID-19 pandemic.

## Introduction

A considerable minority of US adults (approximately 13%)^1,2^ experienced significant increases in distress^2^ during the height of the COVID-19 pandemic. It is not clear whether these increases portend exacerbated or persistent courses of distress, and what risk or protective factors are associated with these courses.

In this study, we build upon our previous study of US military veterans,^2^ which characterized the prevalence of distress (ie, positive screens for major depressive disorder [MDD], generalized anxiety disorder [GAD], or posttraumatic stress disorder [PTSD]) before and 1 year into the COVID-19 pandemic by analyzing 2 additional years of longitudinal data, and identifying factors associated with exacerbated and persistent courses of distress.

## Methods

A total of 2289 US veterans participated in a 3-year longitudinal study as part of the National Health and Resilience in Veterans Study (NHRVS). The study period was divided into 3 phases of the pandemic: prepandemic (Fall 2019), peripandemic (Fall 2020, which included the fall and winter COVID-19 surge before widespread availability of COVID-19 vaccines), and 2-year postonset (Summer 2022, at which point the majority of US adults were vaccinated and mask mandates and social distancing policies no longer enforced). Poststratification weights were employed to yield inferential results representative of the US veteran population. The NHRVS followed the Strengthening the Reporting of Observational Studies in Epidemiology (STROBE) reporting guidelines, and participants provided written informed consent. The ethics committee of VA Connecticut Healthcare System approved the study.

Veterans were categorized into the following groups based on the presence or absence of distress at each time point: *resistant*, signifying participants with no positive screens; *persistent*, for positive screens at each time point; *remitted*, for positive screens at prepandemic only; *resilient*, with positive screen at peripandemic only; or *exacerbated*, with positive screens at peripandemic and 2-year postonset, but not prepandemic) (eTable in Supplement 1). Logistic regression and relative importance analyses examined demographic and clinical factors associated with exacerbated and persistent courses of distress. Data analysis was conducted with SPSS version 27 (IBM). The significance threshold used in regression analyses was *P* < .05.

## Results

Of the total 2289 participating veterans (mean [SD] age, 63.5 [14.0] years; 2057 male participants [92.3%]; 147 [5.8%] Hispanic, 156 [10.4%] non-Hispanic Black, and 1915 [79.7%] non-Hispanic White), most veterans were classified as resistant (1962 participants [83.7%]). The remainder exhibited resilient (105 participants [5.3%]), persistent (96 participants [5.0%]), remitted (78 participants [3.5%]), or exacerbated (48 participants [2.5%]) courses of distress.

In the full sample and all sex and age subgroups except veterans aged 65 years or older, distress significantly increased from the prepandemic to peripandemic period (weighted estimate, 51% increase) but returned to prepandemic levels 2 years later (total positive screens: prepandemic, 174 participants; peripandemic, 249 participants; 2-year postonset, 144 participants) (Table). Veterans aged 18 to 44 years (weighted estimate, 64% increase; 19 positive screens prepandemic vs 29 peripandemic) and female veterans (weighted estimate, 62% increase; 33 positive screens prepandemic vs 52 peripandemic) evidenced the highest peripandemic increases. Results of relative importance analyses revealed that COVID-19 socioeconomic concerns (29.4% relative variance explained [RVE]) and less community integration (28.1% RVE) were the factors with the largest effect sizes for persistent vs remitted distress, while greater prepandemic problematic alcohol use (31.9% RVE) had the largest effect size for exacerbated vs resilient distress (Figure).

**Table.** Prevalence of Positive Screens for MDD, GAD, and PTSD Among US Military Veterans

Characteristic^b^	Positive screens, No. (weighted %)			*P* value^a^		
	Prepandemic	Peripandemic	2-y Postonset	Peripandemic vs prepandemic	2-y postonset vs peripandemic	2-y postonset vs prepandemic
Any positive screen	174 (8.5)	249 (12.8)	144 (7.5)	<.001	<.001	.16
Sex						
Female	33 (15.1)	52 (24.4)	31 (15.1)	.007	<.001	.79
Male	141 (8.0)	197 (11.8)	113 (6.8)	<.001	<.001	.09
Age						
18-44 y	19 (19.0)	29 (31.2)	22 (22.1)	<.001	<.001	.13
45-64 y	82 (10.5)	131 (17.5)	81 (10.1)	<.001	<.001	>.99
≥65 y	73 (4.7)	89 (5.3)	41 (2.4)	.81	<.001	<.001
MDD	102 (5.2)	138 (7.3)	85 (4.3)	<.001	<.001	.11
Sex						
Female	15 (5.2)	20 (8.1)	15 (5.3)	.55	.58	>.99
Male	87 (5.2)	118 (7.2)	70 (4.2)	<.001	<.001	.08
Age						
18-44 y	13 (12.2)	19 (20.5)	15 (12.6)	<.001	<.001	.82
45-64 y	52 (6.8)	68 (8.6)	48 (6.0)	.08	.003	.36
≥65 y	37 (2.4)	51 (3.3)	22 (1.4)	.20	<.001	.04
GAD	80 (4.6)	146 (7.8)	83 (4.3)	<.001	<.001	.92
Sex						
Female	15 (7.6)	31 (16.3)	15 (7.0)	.002	.001	>.99
Male	65 (4.4)	115 (7.1)	68 (4.2)	<.001	<.001	>.99
Age						
18-44 y	15 (16.0)	21 (20.2)	14 (12.5)	.045	<.001	.08
45-64 y	36 (4.9)	82 (11.3)	53 (7.1)	<.001	<.001	.009
≥65 y	29 (1.9)	43 (2.5)	16 (0.9)	.74	<.001	.003
PTSD	87 (3.8)	118 (6.2)	87 (4.7)	<.001	<.001	.04
Sex						
Female	22 (8.1)	27 (10.5)	19 (9.9)	.34	>.99	.39
Male	65 (3.5)	91 (5.8)	68 (4.3)	<.001	<.001	.07
Age						
18-44 y	8 (6.8)	18 (18.3)	17 (16.7)	<.001	.51	<.001
45-64 y	48 (5.4)	64 (8.3)	46 (5.6)	<.001	<.001	.77
≥65 y	31 (2.1)	36 (2.0)	24 (1.5)	>.99	.27	.21

Abbreviations: GAD, generalized anxiety disorder; MDD, major depressive disorder; PTSD, posttraumatic stress disorder.

^a^ McNemar tests were used to evaluate differences between prevalence estimates at each wave.

^b^ Sample sizes for subgroups: female, 232 participants; male 2057 participants; age 18-44 years, 104 participants; age 45-64 years, 676 participants; age ≥65 years, 1509 participants.

**Figure.**
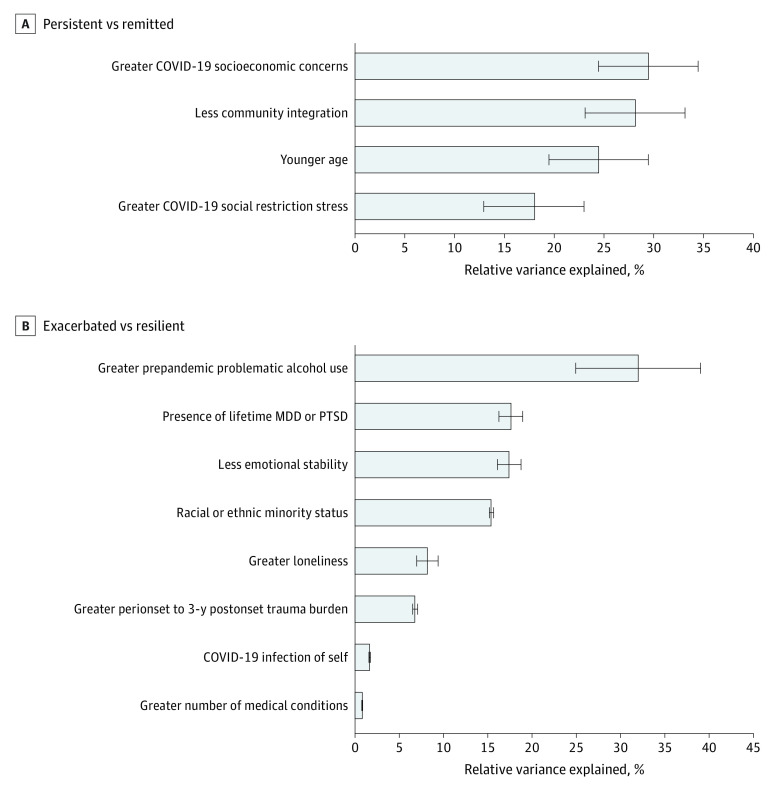
Relative Importance Analysis of Factors Associated With Courses of Distress Error bars represent 95% CIs. Only significant variables from regression models were included in analyses.

## Discussion

This nationally representative longitudinal study of US veterans revealed that, 1 year into the COVID-19 pandemic, the prevalence of positive screens for distress increased by 51%, with younger and female veterans demonstrating the highest increases, possibly due to unique pandemic-related stressors (eg, school closures; work and relationship disruptions).^3^ Nevertheless, 2 years later, distress prevalence returned to prepandemic levels, which aligns with prior work suggesting resilience is the modal response to stressful events.^4^

Despite this overall pattern of resilience, a significant minority of veterans had exacerbated (2.5%; approximately 450 000 based on US veteran population benchmarks) or persistent (5.0%; approximately 900 000) courses of distress. Veterans with exacerbated distress 2 years into the pandemic reported greater prepandemic alcohol use problems, greater likelihood of lifetime MDD or PTSD, and lower emotional stability. Possible mechanisms underlying these findings include alcohol-related neuroinflammatory or metabolic changes^5^ and higher levels of stress sensitization.^6^

Pandemic-related socioeconomic concerns were significantly associated with persistent distress. Younger age, lower community integration, and higher pandemic-related social restriction stress were also associated with this outcome. These findings underscore the importance of assessment, treatment, and policy strategies that target the economic and social needs of veterans with these risk factors.

Limitations of the current study included reliance on self-report and screening measures. Further research is needed to replicate these findings in other samples, identify factors associated with continued distress over time, and evaluate interventions and policies to mitigate distress in veterans at risk for persistent pandemic-related distress.

## Notes

Supplement 1.
eMethods.
**eTable.** Study Measures
eReferences.

Supplement 2.
Data Sharing Statement

## References

[1] Hill ML, Nichter B, Na PJ, . Mental health impact of the COVID-19 pandemic in U.S. military veterans: a population-based, prospective cohort study. Psychol Med. 2021:1-12. doi:10.1017/S0033291721002361 34120667 PMC8245339

[2] Breslau J, Finucane ML, Locker AR, Baird MD, Roth EA, Collins RL. A longitudinal study of psychological distress in the United States before and during the COVID-19 pandemic. Prev Med. 2021;143:106362. doi:10.1016/j.ypmed.2020.106362 33388325 PMC9753596

[3] Lyttelton T, Zang E, Musick K. Telecommuting and gender inequalities in parents’ paid and unpaid work before and during the COVID-19 pandemic. J Marriage Fam. 2022;84(1):230-249. doi:10.1111/jomf.12810 34908583 PMC8661776

[4] Galatzer-Levy IR, Huang SH, Bonanno GA. Trajectories of resilience and dysfunction following potential trauma: a review and statistical evaluation. Clin Psychol Rev. 2018;63:41-55. doi:10.1016/j.cpr.2018.05.008 29902711

[5] Kelley KW, Dantzer R. Alcoholism and inflammation: neuroimmunology of behavioral and mood disorders. Brain Behav Immun. 2011;25(0 1)(suppl 1):S13-S20. doi:10.1016/j.bbi.2010.12.013 21193024 PMC4068736

[6] McLaughlin KA, Conron KJ, Koenen KC, Gilman SE. Childhood adversity, adult stressful life events, and risk of past-year psychiatric disorder: a test of the stress sensitization hypothesis in a population-based sample of adults. Psychol Med. 2010;40(10):1647-1658. doi:10.1017/S0033291709992121 20018126 PMC2891275

