# Supplementary material for: Characterization of Mental Health in US Veterans Before, During, and 2 Years After the Onset of the COVID-19 Pandemic
Source: JAMA Netw Open. 2023 Feb 23;6(2):e230463. doi: 10.1001/jamanetworkopen.2023.0463 (PMC9951035; doi:10.1001/jamanetworkopen.2023.0463)
Supplement: Supplement 1. — eMethods. eTable. Study Measures eReferences. [file jamanetwopen-e230463-s001.pdf]

Supplemental Online Content

Fischer IC, Na PJ, Harpaz-Rotem I, Krystal JH, Pietrzak RH. Characterization of mental health in US veterans before, during, and 2 years after the onset of the COVID-19 pandemic. *JAMA Netw Open.* 2023;6(2):e230463.
doi:10.1001/jamanetworkopen.2023.0463

eMethods.

eTable. Study Measures

eReferences.

This supplemental material has been provided by the authors to give readers additional information about their work.

© 2023 Fischer IC et al. *JAMA Network Open.*

eMethods.

Sample

The National Health and Resilience in Veterans Study (NHRVS) is a nationally representative survey of U.S. veterans. A total of 4,069 veterans completed a Wave 1 (*pre-pandemic*) survey prior to the first documented COVID-19 case in the U.S. (median completion date: 11/21/2019). A total of 3,078 veterans completed a Wave 2 (*peri-pandemic*) survey approximately 1 year later during the 2020 fall/winter surge of COVID-19 cases and before widespread public availability of COVID-19 vaccines (median completion date: 11/14/2020). A total of 2,441 veterans (60% of Wave 1; 79% of Wave 2) also completed a Wave 3 (*2 years into the pandemic*) survey during late summer of 2022 (median completion date: 08/18/2022) when mask-mandates and social distancing policies were no longer enforced, and the majority of US adults had been vaccinated. A comparison of veterans who completed all three survey waves (N=2441) relative to those who did not (N=1628) did not reveal any differences in Wave 1 prevalence of MDD, GAD, or PTSD (all p's>0.24).

The NHRVS sample was drawn from KnowledgePanel, a research panel of more than 50,000 households that is maintained by Ipsos, a survey research firm. KnowledgePanel® is a probability-based, online non-volunteer access survey panel of a nationally representative sample of U.S. adults that covers approximately 98% of U.S. households. Panel members are recruited through national random samples, originally by telephone and now almost entirely by postal mail. Households are provided with access to the Internet and computer hardware if needed. KnowledgePanel® recruitment uses dual sampling frames that include both listed and unlisted telephone numbers, telephone and non-telephone households, and cell-phone-only households, as well as households with and without Internet access.

Demographic data of survey panel members are assessed regularly by Ipsos using the same set of questions used by the U.S. Census Bureau. Race/ethnicity was assessed via self-report using a standard set of questions used by the U.S. Census Bureau; this information was assessed in the current study to characterize the demographic composition of the sample and to adjust for any influence of race/ethnicity in multivariable models.

<u>Ipsos KnowledgePanel Weighting (provided by Ipsos)</u>

Sample Weighting

Significant resources and infrastructure are devoted to the recruitment process for KnowledgePanel so that our active panel members can properly represent the adult population of the U.S. This representation is achieved not only with respect to a broad set of geodemographic indicators, but also for hard-to-reach adults (such as those without Internet access or Spanish-language-dominant Hispanics) who are recruited in proper proportions. Consequently, the raw distribution of KnowledgePanel mirrors that of the U.S. adults fairly closely, barring occasional disparities that may emerge for certain subgroups due to differential attrition. For selection of general population samples from KnowledgePanel, a patented methodology has been developed that ensures all samples behave as EPSEM samples. Briefly, this methodology starts by weighting the pool of active members to the geodemographic benchmarks secured from the latest

© 2023 Fischer IC et al. *JAMA Network Open.*

March supplement of the U.S. Census Bureau's Current Population Survey (CPS) along several dimensions. Using the resulting weights as measures of size, a probability-proportional-to-size (PPS) procedure is used to select study specific samples. It is the application of this PPS methodology with the imposed size measures that produces fully self-weighing samples from KnowledgePanel, for which each sample member can carry a design weight of unity. Moreover, in instances where a study design requires any form of oversampling of certain subgroups, such departures from an EPSEM design are accounted for by adjusting the design weights in reference to the CPS benchmarks for the population of interest.

The geodemographic benchmarks used to weight the active panel members for computation of size measures include:

• Gender (Male/Female)
• Age (18–29, 30–44, 45–59, and 60+)
• Race/Hispanic ethnicity (White/Non-Hispanic, Black/Non-Hispanic, Other/Non-Hispanic, 2+ Races/Non-Hispanic, Hispanic)
• Education (Less than High School, High School, Some College, Bachelor and beyond)
• Census Region (Northeast, Midwest, South, West)
• Household income (under $10k, $10K to <$25k, $25K to <$50k, $50K to <$75k, $75K to <$100k, $100K to <$150k, and $150K+)
• Home ownership status (Own, Rent/Other)
• Metropolitan Area (Yes, No)
• Hispanic Origin (Mexican, Puerto Rican, Cuban, Other, Non-Hispanic)

Study-Specific Post-Stratification Weights
Once all survey data have been collected and processed, design weights are adjusted to account for any differential nonresponse that may have occurred. Given the specific target population this study, geodemographic distributions for the corresponding population are obtained from the Current Veteran Population Supplemental Survey, from the U.S. Census Bureau's American Community Survey (ACS). An iterative proportional fitting (raking) procedure is used to produce the final weights. In the final step, calculated weights are examined to identify and, if necessary, trim outliers at the extreme upper and lower tails of the weight distribution. The resulting weights are then scaled to aggregate to the total sample size of all eligible respondents.

For this study, the following benchmark distributions of U.S. military Veterans from the most recent (August 2019) Current Veteran Population Supplemental Survey data were used for the raking adjustment of weights.

Gender (Male, Female) by Age (18-44, 45-59, 60-69, 70-79, 80+)
• Race/Ethnicity (White/Non-Hispanic, Black/Non-Hispanic, Other/Non-Hispanic, Hispanic, 2+ Races/Non-Hispanic)
• Census Region (Northeast, Midwest, South, West)
• Metropolitan Status (Metro, Non-Metro)
• Education (Less than High School, High School, Some College, Bachelor or higher)

© 2023 Fischer IC et al. *JAMA Network Open.*

• Household Income (under $25K, $25-$49,999, $50K-$74,999, $75K-$99,999, $100K-$149,999, $150K and over)
• Branch of Service (based on self-reported responses to variable service) (Army, Navy, Marine Corps, Air Force, Coast Guard, Other)
• Years in Service (based on self-reported responses to variable Q7) (Less than 2 years, 2 to 3 years, 4-9 years, 10 years and over)

The NHRVS followed the Strengthening the Reporting of Observational Studies in Epidemiology (STROBE) and American Association for Public Opinion Research (AAPOR) reporting guidelines for cohort studies.

American Association for Public Opinion Research Survey Disclosure Checklist:

Survey sponsor	U.S. Department of Veterans Affairs National Center for Posttraumatic Stress Disorder
Survey/Data collection supplier	Ipsos, Inc.
Population represented	U.S. military veterans in the United States
Sample size	2,289
Mode of data collection	Web-based survey panel
Type of sample (probability/non-probability)	Probability
Start and end dates of data collection	November 18, 2019 to September 12, 2022
Margin of sampling error for total sample	+/- 2.57 percentage points at the 95% confidence level
Are the data weighted?	Yes, using the following benchmark distributions of U.S. military veterans from the most recent (August 2019) Current Veteran Population Supplemental Survey of the U.S. Census Bureau's American Community Survey: gender, race/ethnicity, Census Region, metropolitan status, education, household income, branch of service, and years in service
Contact for more information	Robert H. Pietrzak, PhD, MPH<br>Research Psychologist, U.S. Department of Veterans Affairs National Center for PTSD<br>Professor, Department of Psychiatry, Yale School of Medicine<br>(860) 638-7467, robert.pietrzak@yale.edu

© 2023 Fischer IC et al. *JAMA Network Open.*

Analytic plan

Data analysis proceeded in three steps. First, descriptive statistics were calculated to estimate the prevalence of positive screens for MDD, GAD, and PTSD at pre, peri, and 2-years post-onset of the pandemic. (See Supplementary Table 1 for a description of each screening measure.) McNemar's chi-square tests were computed to evaluate differences between prevalence estimates at each wave.

Second, multivariable logistic regression analyses were conducted to examine pre, peri, and 2-years post-onset of the pandemic predictors and correlates of exacerbated and persistent courses of distress. To do so, participants first were categorized into groups based on the presence or absence of a positive screen for MDD, GAD, and/or PTSD at each survey wave. Variables that were significantly associated with these group comparisons at the bivariate level ($p < .01$) were entered as independent variables in the regression analyses. Supplementary Table 1 describes all measures included in bivariate analyses.

Participants were classified as having *persistent* distress if they screened positive for one or more disorders at all three waves (i.e., pre, peri, and 2-years post-onset of the pandemic). Participants were classified as having *remitted* distress if they only screened positive at wave 1. Participants were classified as having *exacerbated* distress if they screened positive for one or more disorders at waves 2 and 3 (i.e., peri and 2-years post-onset of the pandemic) but not wave 1 (pre-pandemic). Participants were classified as *resilient* if they screened positive only at wave 2. Participants were considered to be *resistant* to distress if they screened negative at all three waves. Of the 2,441 participants who completed data at the three waves, 2,289 participants fit into one of these categories. The remaining 152 participants who evidenced different patterns of distress were excluded from analyses.

Third, following these multivariable logistic regression analyses, relative importance analyses[1] were conducted using the R statistical package relaimpo to determine the relative contribution of each significant variable identified in the logistic regression models. These analyses partition the explained variance in the group comparisons that is attributable to each significant independent variable while accounting for intercorrelations among these variables.

© 2023 Fischer IC et al. *JAMA Network Open.*

eTable. Study measures

**Dependent variables**	**Assessment Timepoints: Waves 1, 2, and 3**
Positive screen for MDD	Score ≥3 on the Patient Health Questionnaire-2.[2]
Positive screen for GAD	Score ≥3 on the Generalized Anxiety Disorder-2.[2]
Positive screen for PTSD	Score ≥31 on the PTSD Checklist for DSM-5 was used at Wave 1 and 2.[3] At Wave 3, positive screen for PTSD was assessed using an abbreviated 4-item PCL-5, with a score ≥8 indicative of a positive screen for PTSD.[4]
**Independent variables**	**Assessment Timepoint: Wave 1**
Extraversion	Score on the extraversion subscale of the Ten-Item Personality Inventory.[5]
Agreeableness	Score on the agreeableness subscale of the Ten-Item Personality Inventory.[5]
Openness to experience	Score on the openness to experience subscale of the Ten-Item Personality Inventory.[5]
Conscientiousness	Score on the conscientiousness subscale of the Ten-Item Personality Inventory.[5]
Emotional stability	Score on the emotional stability subscale of the Ten-Item Personality Inventory.[5]
Sociodemographic characteristics	A general sociodemographic questionnaire was used to assess age, sex, race/ethnicity, education, marital status, retirement status, and annual household income.
Military characteristics	
Enlisted/Commissioned vs. Drafted	Were you drafted or did you enlist or earn a commission into the military?
Combat veteran status	"Did you ever serve in a combat or war zone?"
Years in military	"How many years did you spend in the military?"
Physical and mental health characteristics	
Number of medical conditions	Sum of number of medical conditions endorsed in response to question: "Has a doctor or healthcare professional ever told you that you have any of the following medical conditions?" (e.g., arthritis, cancer, diabetes, heart disease, asthma, kidney disease). Range: 0-24 conditions.
Any disability	Any disability in activities of daily living or instrumental activities of daily living. The following questions was asked: "At the present time, do you need help from another person to do the following?" (e.g., bathe; walk around your home or

© 2023 Fischer IC et al. *JAMA Network Open.*

	apartment; get in and out of chair). Endorsement of any of these activities was indicative of having a disability with an activity of daily living. Any disability in instrumental activities of daily living. The following question was asked: "At the present time, do you need help from another person to do the following?" (e.g., pay bills or manage money; prepare bills; get dressed). Endorsement of any of these activities was indicative of having a disability.[6]
Adverse childhood experiences	Score on Adverse Childhood Experiences Questionnaire.[7]
Cumulative trauma burden	Count of potentially traumatic events on the Life Events Checklist for DSM-5 (LEC-5).[8] The LEC-5 was administered at the pre-pandemic, peri-pandemic, and 3-years post-onset assessments, with the latter assessment asking respondents to endorse exposure to potentially traumatic events over the past two years.
Lifetime MDD and/or PTSD	Positive screen for MDD on the Major Depressive Disorder module of the Mini International Neuropsychiatric Interview.[9] Score ≥31 on lifetime version of the PTSD Checklist for DSM-5.[3]
Lifetime AUD and/or DUD	Positive screen for AUD and/or DUD on the alcohol and drug use disorder modules of the Mini Neuropsychiatric Interview for DSM-5,[9] respectively.
Past-year alcohol use severity	Alcohol Use Disorders Identification Test (AUDIT) total score.[10]
Loneliness	The Three-Item Loneliness Scale[11] was used to assess loneliness at the pre-pandemic assessment.
Social support received	Score on the 5-item version of the Medical Outcomes Study Social Support Scale.[12,13]<br>(α = 0.84)
Social support provided	Score on a modified 5-item version of the Medical Outcomes Study Social Support Scale[12,13] that assesses the extent to which an individual provided support to others (e.g., "How often do you provide the following kinds of support to others who need it? – I am someone that helps others with daily chores if they were sick."<br>(α = 0.78)
Community integration	Perceived level of community integration: "I feel well integrated in my community (e.g., regularly participate in community activities)" rating 1=strongly disagree to 7=strongly agree.
Psychosocial and spiritual factors	
Resilience	Score on Connor-Davidson Resilience Scale-10.[14]<br>(α=0.91)

© 2023 Fischer IC et al. *JAMA Network Open.*

Purpose in life	Score on Purpose in Life Test-Short Form.[15] (α=0.89)
Dispositional optimism	Score on single-item measure of optimism from Life Orientation Test-Revised[16]; "In uncertain times, I usually expect the best"); rating 1=strongly disagree to 7=strongly agree.
Dispositional gratitude	Score on single-item measure of gratitude from Gratitude Questionnaire[17]: "I have so much in life to be thankful for" (rating 1=strongly disagree to 7=strongly agree).
Curiosity/exploration	Score on single-item measure of curiosity/exploration from Curiosity and Exploration Inventory-II[18]; "I frequently find myself looking for new opportunities to grow as a person (e.g., information, people, resources") rating 1=strongly disagree to 7=strongly agree.
Religious service attendance	Frequency of attending religious services on Duke University Religion Index.[19]
Private spiritual activities	Frequency of private spiritual activities on Duke University Religion Index.[19]
Intrinsic religiosity	Score on measure of intrinsic religiosity on Duke University Religion Index.[19]
	**Assessment Timepoint: Wave 2**
Cumulative trauma burden	Count of potentially traumatic events on the Life Events Checklist for DSM-5 (LEC-5) that had occurred since Assessment Timepoint Wave 1.[8]
COVID-19 pandemic stressors	Questions from the Coronavirus Health Impact Survey[20] were used to assess COVID-19-associated worries and concerns at the peri-pandemic assessment.<br><br>Factor analysis revealed that these items loaded on five factors (eigenvalues = 1.01–4.94): *COVID-19-related disease worries* (e.g. 'In the past month, how worried have you been about being infected with coronavirus?'); *COVID-19 social restriction stress* (e.g. 'How stressful have these changes in social contacts been for you?'); *COVID-19-associated socioeconomic stress* (e.g. 'In the past month, to what degree have changes associated to the pandemic created financial problems for you or your family?'); *COVID-19-associated relationship difficulties* (e.g. 'Has the quality of the relationships between you and members of your family changed?'); and *COVID-19-associated social engagement* (e.g. 'In the past month, how many people from outside of your household have you had an in-person conversation with?')
	**Assessment Timepoint: Wave 3**

© 2023 Fischer IC et al. *JAMA Network Open.*

Cumulative trauma burden	Count of potentially traumatic events on the Life Events Checklist for DSM-5 (LEC-5) that had occurred since Assessment Timepoint Wave 2.[8]
COVID-19 pandemic stressors	
COVID-19 infection of self	Positive endorsement of "I tested positive for COVID-19."[21]
COVID-19 infection of household member	Positive endorsement of "Someone who lives in my home tested positive for COVID-19?"[21]
COVID-19 infection of non-household member	Positive endorsement of "Someone close to me (but not living in my home) tested positive for COVID-19 (for example, a friend, a family member who does not live with you, a co-worker, or childcare provider)?"[21]
COVID-19 severe infection / hospitalization of self	Positive endorsement of any of the following: "My COVID-19 symptoms were severe enough that there were serious concerns about not surviving;" "My COVID-19 symptoms required a visit to the Emergency Room;" "My COVID-19 symptoms required an overnight stay at a hospital;" "My COVID-19 symptoms required admission to the Intensive Care Unit (ICU);" or "My COVID-19 symptoms were severe enough to require intubation/mechanical ventilation (doctor puts a tube down your throat to help with breathing)."[21]
COVID-19 severe infection / hospitalization of household member	Positive endorsement of any of the following: "Their COVID-19 symptoms were severe enough that there were serious concerns about not surviving;" "Their COVID-19 symptoms required a visit to the Emergency Room;" "Their COVID-19 symptoms required an overnight stay at a hospital;" "Their COVID-19 symptoms required admission to the Intensive Care Unit (ICU);" or "Their COVID-19 symptoms were severe enough to require intubation/mechanical ventilation (doctor puts a tube down your throat to help with breathing)."[21]
COVID-19 severe infection / hospitalization of non-household member	Positive endorsement of any of the following: "Their COVID-19 symptoms were severe enough that there were serious concerns about not surviving;" "Their COVID-19 symptoms required a visit to the Emergency Room;" "Their COVID-19 symptoms required an overnight stay at a hospital;" "Their COVID-19 symptoms required admission to the Intensive Care Unit (ICU);" or "Their COVID-19 symptoms were severe enough to require intubation/mechanical ventilation (doctor puts a tube down your throat to help with breathing)."[21]
Know someone who died from COVID-19	Positive endorsement of "They died of COVID-19" for household and/or non-household member.[21]

© 2023 Fischer IC et al. *JAMA Network Open.*

Abbreviations. MDD=major depressive disorder, PTSD=posttraumatic stress disorder, GAD=generalized anxiety disorder, AUD=alcohol use disorder, DUD=drug use disorder.

© 2023 Fischer IC et al. *JAMA Network Open.*

eReferences.

1. Tonidandel S, LeBreton JM. Relative importance analysis: A useful supplement to regression analysis. Journal of Business and Psychology. 2011;26(1):1-9.
2. Kroenke K, Spitzer RL, Williams JB, et al: An ultra-brief screening scale for anxiety and depression: the PHQ-4. Psychosomatics 2009; 50:613-621.
3. Weathers FW, Litz BT, Keane TM, Palmieri PA, Marx BP, Schnurr PP. The PTSD Checklist for *DSM-5* (PCL-5). 2013. Scale available from the National Center for PTSD at www.ptsd.va.gov.
4. Geier TJ, Hunt, JC, Hanson JL, et al. Validation of abbreviated for-and eight-item versions of the PTSD checklist for DSM-5 in a traumatically injured sample. Journal of Traumatic Stress. 2020;33(3):218-226.
5. Gosling SD, Rentfrow, PJ, Swann Jr, WB. A very brief measure of the Big-Five personality domains. Journal of Research in Personality 37(6):504-528, 2003.
6. Hardy SE, Gill TM: Recovery from disability among community-dwelling older persons. JAMA 291:1596-1602, 2004.
7. Felitti VJ, Anda RF, Nordenberg D, et al: Relationship of childhood abuse and household dysfunction to many of the leading causes of death in adults. The Adverse Childhood Experiences (ACE) Study. Am J Prev Med: 14:245-258, 1998.
8. Weathers FW, Blake DD, Schnurr PP, et al.: The Life Events Checklist for DSM-5 (LEC-5). Instrument available from the National Center for PTSD at www.ptsd.va.gov. 2019.
9. Sheehan DV: Mini International Neuropsychiatric Interview. English Version 7.0.2. For DSM-5. Copyright 1992-2016.
10. Saunders JB, Aasland OG, Babor TF, et al. Development of the alcohol use disorders identification test (AUDIT): WHO collaborative project on early detection of persons with harmful alcohol consumption-II. Addiction. 1993;88(6):791-804.
11. Hughes ME, Waite LJ, Hawkley LC, et al: A short scale for measuring loneliness in large surveys: Results from two population-based studies. Research on aging. 26(6):655-72, 2004.
12. Stewart, AL: The MOS social support survey. Soc Sci Med 32:705-714, 1991.
13. Amstadter AB, Begle AM, Cisler JM, et al: Prevalence and correlates of poor self-rated health in the United States: the national elder mistreatment study. Am J Geriatr Psychiatry 18:615-623, 2010
14. Campbell-Sills L, Stein MB: Psychometric analysis and refinement of the Connor-Davidson Resilience Scale (CD-RISC): validation of a 10-item measure of resilience. J Trauma Stress 20:1019-1028, 2007
15. Schulenberg SE, Schnetzer LW, Buchanan EM. The Purpose in Life test-Short Form: Development and psychometric support. Journal of Happiness Studies 12:861-876, 2011
16. Scheier MF, Carver CS, Bridges MW: Distinguishing optimism from neuroticism (and trait anxiety, self-mastery, and self-esteem): a re-evaluation of the Life Orientation Test. J Pers Soc Psychol 67:1063-1078, 1994
17. McCullough ME, Emmons RA, Tsang J: The grateful disposition: a conceptual and empirical topography. J Pers Soc Psychol 82:112-127, 2002
18. Kashdan TB, Gallagher MW, Silvia PJ, et al: The Curiosity and Exploration Inventory-II: development, factor structure, and initial psychometrics. J Res Pers 2009: 43:987-998.

© 2023 Fischer IC et al. *JAMA Network Open.*

19. Koenig HG, Büssing A: The Duke University Religion Index (DUREL): A five-item measure for use in epidemiological studies. Religions 1:78-85, 2010
20. National Institute of Mental Health. (2020). The CoRonavIruS Health Impact Survey (CRISIS). Retrieved from National Institute of Mental Health Intramural Research Program Mood Spectrum Collaboration, Child Mind Institute of the NYS Nathan S. Kline Institute for Psychiatric Research.
21. National Center for PTSD (NCPTSD) COVID-19 Workgroup. (2021). COVID-19 Exposure Scale. [Unpublished measure]. Available from https: //www.ptsd.va.gov.

© 2023 Fischer IC et al. *JAMA Network Open.*
