# Supplementary material for: Characterization of Mental Health in US Veterans Before, During, and 2 Years After the Onset of the COVID-19 Pandemic
Source: JAMA Netw Open. 2023 Feb 23;6(2):e230463. doi: 10.1001/jamanetworkopen.2023.0463 (PMC9951035; doi:10.1001/jamanetworkopen.2023.0463)
Supplement: Supplement 2. — Data Sharing Statement [file jamanetwopen-e230463-s002.pdf]

Data Sharing Statement

Fischer. Characterization of Mental Health in US Veterans Before, During, and 2 Years After the Onset of the COVID-19 Pandemic. *JAMA Netw Open*. Published February 23, 2023. doi:10.1001/jamanetworkopen.2023.0463

Data
Data available: No

Additional Information
Explanation for why data not available: Veterans Affairs requests persons interested in data reach out directly.
